# Post-error Brain Activity Correlates With Incidental Memory for Negative Words

**DOI:** 10.3389/fnhum.2018.00178

**Published:** 2018-05-08

**Authors:** Magdalena Senderecka, Michał Ociepka, Magdalena Matyjek, Bartłomiej Kroczek

**Affiliations:** ^1^Institute of Philosophy, Jagiellonian University, Kraków, Poland; ^2^Institute of Computer Science and Computational Mathematics, Jagiellonian University, Kraków, Poland; ^3^Berlin School of Mind and Brain, Humboldt-Universität zu Berlin, Berlin, Germany

**Keywords:** emotion, error monitoring, error-related negativity (ERN), event-related potentials (ERPs), incidental memory and learning, incidental recall, post-error positivity (Pe), stop-signal task

## Abstract

The present study had three main objectives. First, we aimed to evaluate whether short-duration affective states induced by negative and positive words can lead to increased error-monitoring activity relative to a neutral task condition. Second, we intended to determine whether such an enhancement is limited to words of specific valence or is a general response to arousing material. Third, we wanted to assess whether post-error brain activity is associated with incidental memory for negative and/or positive words. Participants performed an emotional stop-signal task that required response inhibition to negative, positive or neutral nouns while EEG was recorded. Immediately after the completion of the task, they were instructed to recall as many of the presented words as they could in an unexpected free recall test. We observed significantly greater brain activity in the error-positivity (Pe) time window in both negative and positive trials. The error-related negativity amplitudes were comparable in both the neutral and emotional arousing trials, regardless of their valence. Regarding behavior, increased processing of emotional words was reflected in better incidental recall. Importantly, the memory performance for negative words was positively correlated with the Pe amplitude, particularly in the negative condition. The source localization analysis revealed that the subsequent memory recall for negative words was associated with widespread bilateral brain activity in the dorsal anterior cingulate cortex and in the medial frontal gyrus, which was registered in the Pe time window during negative trials. The present study has several important conclusions. First, it indicates that the emotional enhancement of error monitoring, as reflected by the Pe amplitude, may be induced by stimuli with symbolic, ontogenetically learned emotional significance. Second, it indicates that the emotion-related enhancement of the Pe occurs across both negative and positive conditions, thus it is preferentially driven by the arousal content of an affective stimuli. Third, our findings suggest that enhanced error monitoring and facilitated recall of negative words may both reflect responsivity to negative events. More speculatively, they can also indicate that post-error activity of the medial prefrontal cortex may selectively support encoding for negative stimuli and contribute to their privileged access to memory.

## Introduction

Recent years have produced many studies investigating the interaction between emotion and error monitoring. Most of these reports focused on the long-lasting negative affect associated with psychiatric diseases or character traits (for reviews, see Vaidyanathan et al., 2012; Endrass and Ullsperger, 2014). A relatively small number of works have examined the influence on performance monitoring of short-duration affective states induced by emotional stimuli, such as pictures, film clips or sounds (e.g., Larson et al., 2006; van Wouwe et al., 2010; Senderecka, 2018). However, no study has tested whether processing of emotional words can lead to increased error detection. In addition, although it seems reasonable to assume that emotional modulation of error monitoring may be associated with more efficient encoding of affective material and its subsequent recall from memory, the link between these effects has not been yet explored. The aim of the present study was to fill these gaps by investigating the links between short-duration affective states induced by emotional words, error monitoring and incidental memory.

Thus, our study had three specific goals. First, we aimed to evaluate whether short-duration affective states induced by emotional words can enhance error monitoring, as reflected by electrophysiological indices. Second, we intended to assess whether such an enhancement (if present) is specific to unpleasant or pleasant linguistic stimuli, or is a general response to arousing material, irrespective of valence. Third, we decided to examine whether post-error brain activity correlates with incidental memory for negative and/or positive words. To reach these goals, we used behavioral measures, as well as event-related potential (ERP) components.

Error monitoring is defined as the ability to evaluate ongoing actions, detect an error and dynamically adjust performance, which is critical to the adaptive control of behavior in a frequently changing environment. It constitutes part of a larger cognitive control system and is primarily related to activity in the medial frontal cortex (Ridderinkhof et al., 2004). The electrophysiological signature of error monitoring is reflected in two components of scalp-recorded ERP: error-related negativity (ERN; Gehring et al., 1993), also called error negativity (Ne; Falkenstein et al., 1990), and post-error positivity (Pe; Falkenstein et al., 1991).

Error-related negativity is a sharp, negative deflection that occurs over the fronto-central regions and peaks at around 0–100 ms after the commission of an error (Falkenstein et al., 1990; Gehring et al., 1993). Various theories about the functional significance of ERN point to different possibilities, ranging from a mechanism that monitors the difference between an intended and an actually performed action (Falkenstein et al., 1991; Coles et al., 2001), through to the result of a conflict between simultaneously active correct and incorrect response tendencies (Botvinick et al., 2001; Yeung et al., 2004), and a signal of reinforcement learning (Holroyd and Coles, 2002). More recent findings suggest that ERN reflects an increase in attentional control, supported by enhanced activation of the medial frontal cortex, typically observed in situations demanding monitoring of ongoing actions (van Noordt et al., 2015, 2016, 2017). Meanwhile, other studies strongly indicate that the ERN amplitude reflects the subjective significance of an error (Gehring et al., 1993; Hajcak et al., 2005) or the accompanying negative affect and emotional distress (Hajcak and Foti, 2008; Inzlicht and Al-Khindi, 2012).

A second ERP component related to error monitoring, namely the Pe, is a positive wave that is more sustained than ERN and occurs over the centro-parietal regions, approximately between 100 and 400 ms after error commission (Falkenstein et al., 1991). As in the case of ERN, several accounts have been proposed regarding its functional significance. A large body of research suggests that the Pe is associated with conscious recognition of an error and increased awareness of performance abilities (Nieuwenhuis et al., 2001; Endrass et al., 2007; Larson and Perlstein, 2009; Hughes and Yeung, 2011). Other studies indicate that the Pe displays topographical similarities to the stimulus-related P3 and may thus reflect the increased motivational significance of erroneous responses, which are rare, distinctive and salient events (Leuthold and Sommer, 1999; Ridderinkhof et al., 2009; Endrass et al., 2012). This is in line with the assumption that the Pe may be a manifestation of the emotional appraisal of an error or its consequences (Falkenstein et al., 2000). Additionally, the Pe is also considered an index of the accumulation of evidence that an error has occurred (Steinhauser and Yeung, 2010; see also Ullsperger et al., 2010; Wessel et al., 2011).

Research in the last two decades has yielded a substantial body of evidence showing that long-lasting negative affect and emotional distress are usually accompanied by increased error monitoring. For example, an enhanced ERN amplitude has been demonstrated in patients with anxiety disorders (Hajcak et al., 2003; Aarts and Pourtois, 2010) and obsessive–compulsive disorder (Gehring et al., 2000; Endrass et al., 2008, 2010; for a review, see Endrass and Ullsperger, 2014). A reliable increase of ERN has also been observed among non-clinical individuals with high levels of negative affect (Luu et al., 2000; Hajcak et al., 2004). Some studies have also reported enhanced performance monitoring, as indexed by ERN amplitude, among patients suffering from major depression (Chiu and Deldin, 2007; Holmes and Pizzagalli, 2008, 2010). Meanwhile, however, other studies have indicated that severe depression may also result in reduced ERN (Olvet et al., 2010, for a review, see Vaidyanathan et al., 2012).

While much attention has been paid to the relationship between error monitoring and long-lasting affective states, relatively less has been paid to the impact of short-term changes in emotion on error monitoring. Two previous studies have measured ERN in the context of viewing emotional pictures used to induce short-duration affective states (Larson et al., 2006; Wiswede et al., 2009a). Larson et al. (2006) observed enhanced ERN amplitude on trials in which flanker stimuli were superimposed on positive pictures, whereas Wiswede et al. (2009a) found increased ERN on flanker trials that followed the presentation of negative pictures. In turn, van Wouwe et al. (2010) observed enhanced ERN in individuals who viewed a positive film clip before being asked to complete a continuous performance task. In addition, increased ERN was reported in studies that used more abstract emotional manipulation to investigate whether error monitoring is influenced by derogatory verbal feedback, motivational impact of punishment, or induction of feelings of helplessness (Wiswede et al., 2009b; Riesel et al., 2012; Pfabigan et al., 2013). However, it is worth noting that some studies failed to find ERN amplitude modulation in response to fear or sad and happy mood induction (Moser et al., 2005; Paul et al., 2017). Importantly, using a spatial Stroop task, Ogawa et al. (2011) found reduced ERN in the condition in which erroneous responses were followed by verbal admonishment. In summary, the studies reviewed above are extremely difficult to integrate due to the substantial variability of methodology, leading to contrary results and conclusions. Furthermore, although there is sparse evidence that points to the influence of short-duration affective states on the Pe amplitude (Moser et al., 2005; Paul et al., 2017), in the majority of these studies only the first component of the ERN-Pe error-related complex was taken into consideration.

Recently Senderecka (2016, 2018) investigated the influence of emotional visual and auditory stimuli on both error-related components simultaneously in a stop-signal paradigm. Participants performed an emotional stop-signal task (SST) that required response inhibition to briefly presented aversive and neutral pictures or sounds. The analyses revealed that negative stimuli from both sensory modalities improved error monitoring by increasing the Pe amplitude. However, the ERN amplitude was comparable in the emotional and neutral conditions, which agreed with some earlier studies (Moser et al., 2005; Paul et al., 2017), but contrasted with others (Larson et al., 2006; Wiswede et al., 2009a,b; van Wouwe et al., 2010; Ogawa et al., 2011; Riesel et al., 2012; Pfabigan et al., 2013). Given the inconsistency among methodological approaches and the discrepancy in the findings reviewed above, it can be stated that there is a clear need for systematic examination of both error-related components in a series of related and similarly designed tasks. Thus, the present study aimed to expand on Senderecka (2016, 2018) by further exploring the mechanism of the emotional enhancement effect on error monitoring in the SST paradigm.

The first goal of the present study was to test whether the previous pattern of results, which points to the emotional enhancement of error detection in the SST, could be obtained with linguistic stimuli. To address this question, we used an SST requiring response inhibition to negative, positive and neutral nouns. Most of the words are entirely symbolic signs whose meaning is acquired by learning. Thus, responses to such stimuli are not based on biological predisposition and have not been shaped by evolutionary pressures, unlike responses evoked by emotional pictures and sounds, especially aversive ones (Öhman and Mineka, 2001). In line with these considerations, there is broad agreement that emotional linguistic stimuli are less arousing than other types of visual affective material such as emotional scenes or facial expressions (Vanderploeg et al., 1987; Keil, 2006; Kissler et al., 2006; Hinojosa et al., 2009). Indeed, some studies revealed that, contrary to what occurs with affective pictures (Vuilleumier et al., 2001; Schimmack and Derryberry, 2005; Verbruggen and De Houwer, 2007), emotional words are, in general, not capable of interfering with performance in ongoing cognitive tasks in healthy participants, probably because of the limited arousing power of linguistic material (for reviews, see Williams et al., 1996; Siegle et al., 2002). On the other hand, a growing body of studies indicates that arousing words are able to attract enhanced attention compared to neutral words and to influence cognitive processing across a number of experimental tasks (e.g., Carretié et al., 2008; Chiu et al., 2008; Estes and Verges, 2008; Kanske and Kotz, 2010; Herbert and Sütterlin, 2011). These latter findings clearly suggest that the emotional intensity of linguistic stimuli is associated with the degree of interference caused by them in the ongoing cognitive task. Thus, the present study was intended to further examine this association through the analysis of ERP correlates of error monitoring, using SST with linguistic stimuli.

The second goal of the study was to test whether emotional enhancement of error monitoring (if present) is limited to words of specific valence. Current emotional state is modulated by both valence and arousal, two affective dimensions that are widely considered to explain the variance in emotional salience (Lang et al., 1990, 1993; Lang, 1995). Valence reflects how the motivational system responds to a stimulus (either appetitively or aversively), whereas arousal reflects the intensity of its reaction. In our previous studies (Senderecka, 2016, 2018) the emotional salience of stop signals was manipulated using either threatening and neutral pictures, or aversive and neutral sounds. Thus, the positively valenced stimuli were not included in the task. For this reason, it remains unclear whether the observed emotional modulation of error monitoring in the SST was specifically related to negative valence or rather to high arousal of unpleasant stop-signals. Given that two previous flanker studies (Larson et al., 2006; Wiswede et al., 2009a) which examined error detection in the context of viewing negative and positive pictures produced divergent results (selectively increased ERN either in the negative or positive condition), it is still unknown which affective dimension is a determinant for the strength of the emotional influence on performance monitoring. Thus, if there are emotion-based changes in error detection in the SST, it would be beneficial to explore whether they can be evoked by stimuli from both affective valence categories.

The third goal of the present study was to determine whether post-error brain activity correlates with incidental memory for emotional words. Incidental memory refers to the ability to encode and maintain information without prior intention to remember (Rugg et al., 1997). Emotions exert powerful influences on learning and memory that involve different brain systems engaged at multiple stages of information processing (LaBar and Cabeza, 2006). The findings from previous studies suggest that memory for emotional words is better than for neutral words (e.g., Herbert et al., 2008a,b; Ferré et al., 2015). For instance, negative words such as *death* are more likely to be recalled than neutral words such as *bottle* (Rubin and Friendly, 1986). The memory advantage of emotional over neutral information is called the *emotion-enhanced memory* effect (EEM, Hamann et al., 1999; Talmi et al., 2007). Emotional valence/arousal effects of linguistic stimuli can be seen in memory performance, even when the meaning of the experimental stimuli is processed incidentally (Kissler et al., 2007, 2009; Herbert et al., 2008a,b; but see also Ramponi et al., 2010). Additionally, results indicate that one valence might affect memory performance differently than another (for a review, see Bowen et al., 2017), suggesting that memory improvement might be valence-specific.

The EEM occurs in tasks involving a long delay between an initial study phase and a later memory test, as well as in immediate free-recall memory tests, i.e., those using retention intervals of several minutes (for a review, see Murty et al., 2010). It has been suggested that in studies with long retention intervals, the EEM is primarily due to a better consolidation of emotional memory traces than that of neutral stimuli (McGaugh, 2004; Phelps, 2004). In turn, in studies with short retention intervals the EEM probably relies on a different mechanism (Talmi et al., 2007), if only because the delay between encoding and retrieval is too small to allow consolidation to occur (McGaugh, 2004). The memory improvement for affective material observed on immediate recall or after short delays may be due to multiple factors that play a significant role during encoding, such as enhanced perceptual sensitivity (Zeelenberg et al., 2006) or increased physiological arousal (LeDoux, 2000). A growing body of studies indicates that the EEM may also be a result of increased involvement of attention during encoding (Hamann, 2001; Calvo and Lang, 2004; Talmi and McGarry, 2012). Importantly, the increase in attentional control is also typically observed in situations demanding ongoing monitoring of performance (van Noordt et al., 2015, 2016, 2017). This raises the question of whether the increased involvement of attention during error detection is associated with more efficient memory performance for emotional stimuli. We can tentatively assume that error monitoring may provide an additional source of modulation for the processing of affective stimuli that may ultimately contribute to their privileged access to awareness and memory. Thus, it seems reasonable to ask whether post-error brain activity correlates with the strength of immediate recall for affective material presented within the task. Such a correlation analysis can provide important knowledge about memory performance in an error-monitoring context. To our knowledge, the link between these two mechanisms has not been explored yet.

The present study’s hypothesis is that emotional words induce transient affective states which dynamically modulate error monitoring. We predicted that the response-locked Pe component would show increased amplitude in both negative and positive conditions. Based on our previous results (Senderecka, 2016, 2018), emotional enhancement of ERN amplitude was not expected. Finally, we assumed that post-error brain activity would be associated with incidental memory performance for emotional words.

## Materials and Methods

### Participants

Sixty-five volunteers (39 females and 26 males) aged 18–34 years old (*M* = 23.7 years, *SD* = 4.2) were recruited via Internet advertisements and were paid the equivalent of about 5 US dollars in Polish zloty (PLN). All participants were in good health, free of medications, and had normal or corrected-to-normal vision. None reported a history of psychiatric or neurological diseases. Of the initial sample recruited for the study, three participants were excluded from the analyses because they turned out not to be native speakers of Polish; two participants were excluded because of technical problems with the EEG recording or excessive EEG artifacts; one participant was excluded due to a probable misunderstanding of the instructions which led to an extremely small amount of correct responses; and another one was excluded because his mean RT deviated substantially from the mean of the sample (more than +3.0 standard deviations). The remaining 58 participants (33 females and 25 males), 18–34 years old had a mean age of 23.4 years (*SD* = 3.9). The sample size was determined based on literature (Steele et al., 2016), our previous studies (Senderecka, 2016, 2018) and power analysis. The results indicated that our sample size would allow detection of a moderate effect size (*f* = 0.15) with a power >80%, at an alpha level of 0.05 (Cohen, 1988).

### Stimuli

Stimuli consisted of 81 words selected from the Nencki Affective Word List (NAWL; Riegel et al., 2015; Wierzba et al., 2015), which has recently been introduced as a standardized database of Polish words suitable for studying various aspects of language and emotions. The stimulus set contained 27 negative (e.g., anger, death, punishment), 27 positive (e.g., love, miracle, promotion), and 27 neutral (e.g., feature, product, document) nouns. Normative ratings indicated that negative words were less pleasant [*t*(26) = 32.84, *p* < 0.001, *d* = 1.83] than neutral words, which were less pleasant [*t*(26) = 32.32, *p* < 0.001, *d* = 2.00] than positive words. Both negative [*t*(26) = 54.62, *p* < 0.001, *d* = 2.00] and positive [*t*(26) = 31.47, *p* < .001, *d* = 2.10] words were more emotionally arousing than neutral words. However, normative ratings of arousal for negative and positive words were not significantly different from one another [*t*(26) = 0.50, *p* = 0.62, *d* = 0.50]. Specific words used in the study appear in Supplementary Table S1. Stimulus categories were controlled regarding word frequency, word length (numbers of letters and syllables) and imageability ratings, all *Fs* (2,52) < 1; for the words’ characteristics, see **Table 1**.

**Table 1:** Words characteristics.

	Negative	Positive	Neutral
Valence (-3 to 3)	-2.1 (0.3)	2.3 (0.2)	0.1 (0.3)
Arousal (1 to 5)	3.4 (0.2)	3.5 (0.3)	1.4 (0.1)
Imageability (1 to 7)	5.8 (0.3)	5.9 (0.6)	5.8 (0.6)
Frequency^a^	55.2 (76.5)	54.4 (52.4)	53.9 (66.7)
Number of letters	6.6 (2.0)	6.8 (2.0)	6.9 (2.1)
Number of syllables	2.3 (1.0)	2.2 (0.7)	2.5 (0.9)


*^*a*^Frequency measured as the number of occurrences per million words.*

### Procedure and Task

The experimental procedure was in accordance with the ethical principles of the 1964 Declaration of Helsinki (World Medical Organization, 1996) and conformed to the ethical guidelines of the National Science Centre of Poland (2016). The protocol was approved by the Research Ethics Committee at the Philosophical Faculty of the Jagiellonian University in Kraków, Poland. Participants were seated in a dimly lit, sound-attenuated, air-conditioned testing room. After providing written informed consent to participate in the study, they completed the SST with emotional and neutral words. They were asked to restrict body movements and blinking as much as possible during the recording of the EEG. Immediately after the SST, they were instructed to write on a blank sheet of paper all the words they could remember from those presented during the task.

The SST required participants to perform a primary binary-choice response task. Each trial began with the presentation of a black central fixation cross for 800 ms, immediately followed by the presentation of the go stimuli. Two black arrows pointing left or right served as these stimuli. They were presented randomly one at a time, for 100 ms, each with 50% probability, on a gray background in the center of a 23″ computer monitor. Participants were instructed to respond by pressing the left or right “ctrl” key on a computer keyboard according to the direction of the arrow that was presented to them. If the arrow pointed to the left, they were to respond by pressing the left “ctrl” key using their left index finger; if the arrow pointed to the right, they were to respond by pressing the right “ctrl” key using their right index finger. In addition, they were asked to react to the go stimuli as quickly and accurately as possible.

In a random sample of 25% of the trials, an emotionally negative, positive or neutral noun followed the go stimuli for 1300 ms (in successfully inhibited trials) or until the participant’s response (in unsuccessfully inhibited trials), serving as the stop signal. The words subtended between 2.6 and 7.9 of visual angle horizontally when presented onscreen at a comfortable viewing distance of approximately 65 cm, in front of the participant, at eye level. Participants were instructed to inhibit their response while viewing a word that followed the initial go stimulus, regardless of which arrow was presented. They were also told that sometimes it might not be possible to successfully inhibit their response and that in such cases they should simply continue performing the task. Overall, the importance of going and stopping was stressed equally.

**FIGURE 1:**
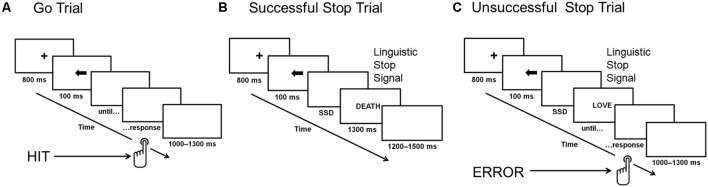
Stop-signal task. Panel **(A)** presents a go trial without stop-signal presentation, panel **(B)** shows a successfully inhibited stop-signal trial, and panel **(C)** illustrates an unsuccessfully inhibited stop-signal trial. ERROR, unsuccessfully inhibited response; HIT, correct response to go stimuli; SSD, stop-signal delay.

Each word occurred two times during the study. A tracking method was used to vary the interval between the presentation of the go stimulus and the stop-signal (i.e., the stop-signal delay, SSD): the interval increased or decreased by 50 ms (from 100 to 400 ms) for the next stop-signal trial, depending on whether the participants successfully inhibited or failed to inhibit their response to the go stimulus in the previous stop-signal trial. Thus, there were seven possible SSDs: 100, 150, 200, 250, 300, 350, and 400 ms. After a successful inhibition, the inter-stimulus interval became longer (thereby making inhibition more difficult on a subsequent stop-signal trial); after an unsuccessful inhibition, it became shorter (making inhibition easier on a subsequent stop-signal trial). The initial value of the SSD was set to 150 ms. The staircasing was done separately for three stop-signal conditions to ensure successful inhibition in approximately 50% of the stop trials in each condition. **Figure 1** presents an outline of the SST design.

Participants received one practice block of 40 trials before data collection to familiarize themselves with the task. In this training run we used a separate set of neutral words as stop signals. After the practice run, participants completed eight experimental blocks, each consisting of 81 trials, with short breaks between blocks. The trial order was randomized with the restriction that any given two stop trials had to have at least one go trial between them. The task was implemented using PsychoPy software (Peirce, 2007).

### EEG Recording

The continuous scalp electroencephalogram (EEG) was recorded from 32 silver/silver-chloride (Ag/AgCl) active electrodes (with preamplifiers) using the BioSemi Active-Two system: Fp1/Fp2, AF3/AF4, F3/F4, F7/F8, FC1/FC2, FC5/FC6, T7/T8, C3/C4, CP1/CP2, CP5/CP6, P3/P4, P7/P8, PO3/PO4, O1/O2, Fz, Cz, Pz, Oz. The electrodes were secured in an elastic cap (Electro-Cap), according to the extended 10–20 international electrode placement system. The signal was continuously recorded at 256 Hz and referenced online to the CMS-DRL ground, which drives the average potential across all electrodes as close as possible to amplifier zero. Electrode offsets were kept within a range of ±20 μV. The horizontal and vertical electro-oculograms (EOGs) were monitored using four additional electrodes placed above and below the right eye and in the external canthi of both eyes. The electrical signal was not filtered during EEG acquisition. All channels were re-referenced off-line to the average of the two mastoid electrodes. The recordings were filtered off-line with a high-pass filter of 0.05 Hz (slope 24 dB/oct) and a low-pass filter of 25 Hz (slope 12 dB/oct). Ocular and other stationary artifacts were removed with the independent component analysis (ICA) algorithm using the Brain Vision Analyzer 2 (Brain Products, Munich, Germany).

### Data Quantification

Response-locked (-100 to 600 ms relative to the key press) segments were subsequently checked and averaged. Contaminated trials exceeding maximum/minimum amplitudes of ±65 μV were rejected by a semi-automatic procedure. The mean number of rejected trials was low (1.9% on average).

**FIGURE 2:**
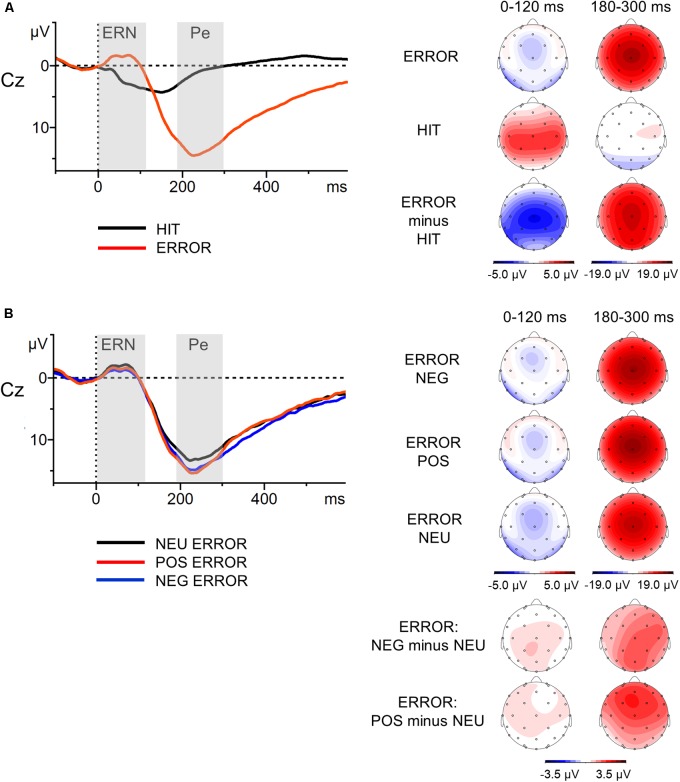
Response-locked grand-average waveforms at Cz electrode (left part) with scalp potential maps for the ERN and Pe components (right part). Panel **(A)** presents grand-average ERPs for erroneous and correct response trials and topographic maps for the Error, Hit and Error-minus-Hit-difference waves. Panel **(B)** illustrates grand-average ERPs to the erroneous negative, erroneous positive and erroneous neutral responses, and topographic maps for the NEG/POS/NEU Error waves and two difference waves: NEG-minus-NEU Error and POS-minus-NEU Error. The component-specific windows examined in this study are highlighted. Error, unsuccessfully inhibited responses; Hit, correct responses to go stimuli; NEG, negative; NEU, neutral; POS, positive.

Motor reaction ERPs were calculated separately for correct (Hit) and unsuccessfully inhibited (Error) responses. In addition, grand averages for incorrect responses were calculated separately for erroneous responses following negative (NEG Error), positive (POS Error), and neutral (NEU Error) stop-signal presentations. The mean number of correct, artifact-free epochs included in the ERP analysis across all participants for each of the response trial categories were as follows: Hit *M* = 477.1 (*SD* = 12.8); Error *M* = 77.7 (*SD* = 9.8); NEG Error *M* = 25.9 (*SD* = 3.1); POS Error *M* = 25.6 (*SD* = 3.7); NEU Error *M* = 26.2 (*SD* = 3.8). The minimum number of epochs was 397 for Hit, 38 for Error, 16 for NEG Error, 13 for POS Error, 9 for NEU Error. Thus, error-related components were based on no fewer than nine artifact-free error trials, a number that is sufficient to achieve stable estimates of the ERN and Pe (Olvet and Hajcak, 2009; Steele et al., 2016). Consistent with previous research on the error-related ERP components in the SST paradigm (Beyer et al., 2012), we focused on electrode Cz, where these components were found to be highest (see topographic maps in **Figure 2A**). In line with the literature (van Veen and Carter, 2002; Fiehler et al., 2005; Ullsperger and von Cramon, 2006), the mean voltage amplitudes in the post-response time-windows of 0–120 ms (ERN) and 180–300 ms (Pe) were selected. ERPs were baseline-corrected relative to the pre-response interval from -100 to 0 ms.

### Statistical Analyses

To compare inhibitory performance across the three stop-signal conditions (negative, positive and neutral), two one-way repeated-measures ANOVAs were conducted on the behavioral variables: stop-signal reaction time (SSRT) and inhibition rate. The SSRT, which provides an estimate of the latency of the inhibitory process, was calculated following the procedure of Logan (1994). Reaction times from go stimuli responses in which no stop signal occurred were collapsed into a single distribution and rank ordered. The *n*th reaction time was selected, where *n* was obtained by multiplying the number of no-signal reaction times in the distribution (486) by the probability of responding (e.g., 0.5 if the global inhibition rate was equal to 50%) for each participant separately. The global SSRT was calculated by subtracting the average SSD from the *n*th reaction time (RT), following the horse race model (see Logan and Cowan, 1984; Verbruggen and Logan, 2008 for more detail). In turn, the SSRTs for each stop-signal condition were calculated by subtracting the negative/positive/neutral SSD from the *n*th reaction time, chosen based on the condition-wise probability of responding.

To analyze the amplitudes of ERN and the Pe, two one-way repeated-measures ANOVAs were conducted (separately for each component): the first with the Response Type (Hit, Error), and the second with the Error Condition (NEG Error, POS Error, NEU Error) as factors. All continuous variables were examined with the Kolmogorov–Smirnov test; this showed that the distributions of the variables were not statistically different from the normal distribution, except for percentages of correctly recalled negative, positive and neutral words, which were thus log-transformed for Pearson correlation analysis. The critical *p* value was set at.05 for all the analyses. To interpret significant findings, global analyses were followed by restricted *post hoc t*-tests.

## Results

### Behavioral Data

Behavioral results are summarized in **Table 2**. Only correct trials (>99%) were taken into consideration in the mean RT analyses of the go trials. In order to control for outliers, trials on which RT was more than 3.0 standard deviations above or below the participant’s mean RT were excluded from the behavioral analysis (1.2% of trials). The mean RT of the correct go trials was 431.0 ms (*SD* = 58.1). The global SSRT was 206.3 ms (*SD* = 29.7), whereas the global SSD was 213.8 ms (*SD* = 54.4).

**Table 2:** Behavioral results – means (standard deviations).

Go performance
Go RT (ms)	431.0 (58.1)		
Go error rate (%)	0.7 (0.8)		

**Stop performance**	**Negative**	**Positive**	**Neutral**

Inhibition rate (%)	50.6 (5.1)	50.5 (5.1)	50.1 (5.8)
SSD (ms)	215.0 (55.8)	213.7 (54.5)	212.7 (56.8)
SSRT (ms)	205.9 (29.4)	207.3 (33.0)	209.4 (33.9)

**Memory performance**			

Correctly recalled words (%)	14.3 (7.9)	14.3 (7.4)	6.4 (5.3)


*SSD, stop-signal delay; SSRT, stop-signal reaction time; RT, reaction time.*

As expected because of the staircasing procedure, stop performance was approximately 50% correct in all three conditions (negative: 50.6%, positive: 50.5%, neutral: 50.1%) and no main effect of emotion was observed in the repeated-measures ANOVA analysis [*F*(2,114) = 0.79, *p* = ns]. The SSRT did not differ significantly between the three stop-signal conditions [*F*(2,114) = 0.58, *p* = ns], indicating that stop performance was comparable in the emotional and in the neutral stop-signal trials. The SSD was also comparable in all conditions [*F*(2,114) = 0.33, *p* = ns].

Incidental recall was superior for emotional relative to neutral words [*F*(2,114) = 41.70, *p* < 0.001, $\eta_{p}^{2}$ = 0.42]. This was true both for negative words [*t*(57) = 6.70, *p* < 0.001, *d* = 0.98] and positive words [*t*(57) = 7.86, *p* < 0.001, *d* = 1.07]. Correct recall did not differ between negative and positive words [*t*(57) = 0.35, *p* = ns].

### ERP Findings

The results of the global analysis conducted on both components are presented in the upper part of **Table 3**; the mean amplitudes and standard deviations for two components in all experimental conditions are shown in the lower part of **Table 3**. **Figure 2** presents the grand-average ERPs to the motor reaction at Cz with scalp distribution maps for difference waves.

**Table 3:** Results of the global analysis of the ERP components.

Component	Effect	*F*	*p*	$\eta_{p}^{2}$
	Response Type^a^ (Hit and Error)			
ERN		38.46	<0.001	0.40
Pe		209.41	<0.001	0.79
	Error Condition^b^ (NEG, POS, and NEU)			
ERN		0.78	=0.46	0.01
Pe		6.34	=0.002	0.10

**Components’ mean amplitude results (μV)**		**ERN**	**Pe**	

Hit		2.1 (2.8)	1.3 (4.7)	
Error		-0.7 (4.0)	13.3 (5.2)	
NEG Error		-0.5 (4.5)	13.7 (6.1)	
POS Error		-0.7 (4.4)	14.0 (5.4)	
NEU Error		-1.0 (4.3)	12.4 (5.2)	


*Error, unsuccessfully inhibited responses; Hit, correct responses to go stimuli; NEG, negative; NEU, neutral; POS, positive; ^*a*^*df* = 1,57; ^*b*^*df* = 2,114.*

#### ERN Component (0–120 ms)

The global analysis revealed that the main effect of Response Type was significant [*F*(1,57) = 38.46, *p* <0.001, $\eta_{p}^{2}$ = 0.40]. The ERPs to Error (unsuccessfully inhibited response, time-locked to the button press) showed a sharp negative peak which was attenuated in the ERPs to Hit (Δ*M* = 2.8 μV). The ERN amplitudes were statistically comparable [*F*(2,114) = 0.78, *p* = 0.46, $\eta_{p}^{2}$ = 0.01) in the NEG, POS and NEU Error trials (all Δ*M* ≤ 0.5 μV).

#### Pe Component (180–300 ms)

The ERPs to Error displayed sustained positive activity (following the ERN) which was absent in the ERPs to Hit (Δ*M* = 12.0 μV). Thus, the ANOVA showed a main effect of Response Type [*F*(1,57) = 209.41, *p* < 0.001, $\eta_{p}^{2}$ = 0.79]. Statistical analysis revealed that the main effect of Error Condition was also significant [*F*(2,114) = 6.34, *p* = 0.002, $\eta_{p}^{2}$ = 0.10]. The Pe amplitudes time-locked to the motor reaction in the emotional Error trials were greater than in the NEU Error trials. This was true for both NEG Error trials [*t*(57) = 2.75, *p* = 0.008, *d* = 0.23, Δ*M* = 1.3 μV] and POS Error trials [*t*(57) = 3.36, *p* = 0.001, *d* = 0.30, Δ*M* = 1.6 μV]. The Pe amplitudes did not differ between NEG and POS Error trials [*t*(57) = 0.54, *p* = ns, Δ*M* = 0.3 μV].

### Correlation Analyses

**FIGURE 3:**
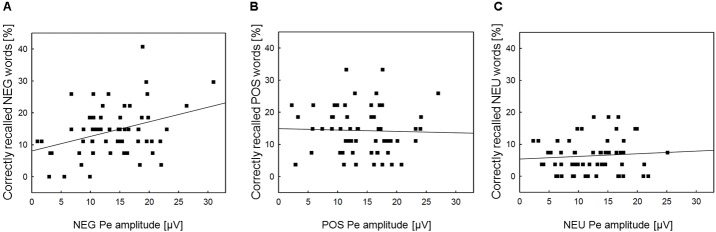
Scatterplots and regression lines within emotion categories. Panel **(A)** presents the relationships between the Pe amplitude in NEG erroneous response trials and incidental recall performance for negative words. Panel **(B)** shows the relationships between the Pe amplitude in POS erroneous response trials and incidental recall performance for positive words. Panel **(C)** illustrates the analogous association between the Pe amplitude in NEU erroneous response trials and incidental recall performance for neutral words. NEG, negative; NEU, neutral; POS, positive.

Correlation analyses were performed to explore associations between memory processing and the Pe component, for which emotional enhancement effects were observed. We intended to check whether the increased Pe amplitude in the two emotional error conditions is associated with facilitated incidental recall for emotional words. The Pearson correlation analyses revealed that memory performance for negative words was significantly correlated with the Pe amplitude in the NEG Error trials (*r* = 0.35, *p* = 0.007)^1^. However, there was no significant correlation either between memory performance for positive words and the Pe amplitude in the POS Error trials (*r* = -0.04, *p* = ns), or between memory performance for neutral words and the Pe amplitude in the NEU Error trials (*r* = 0.04, *p* = ns). **Figure 3** shows scatterplots revealing how Pe amplitudes in negative, positive and neutral error conditions were associated with incidental recall for words from the corresponding category.

**FIGURE 4:**
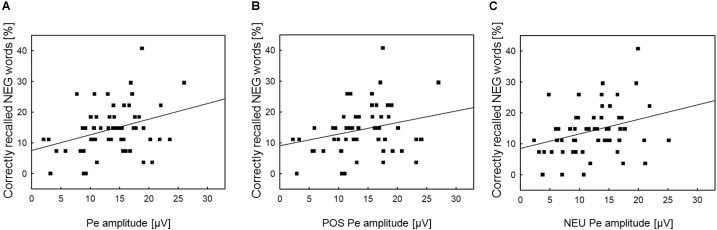
Scatterplots and regression lines across emotion categories. Panel **(A)** presents the relationships between the Pe amplitude in erroneous response trials and incidental recall performance for negative words. Panel **(B)** shows the relationships between the Pe amplitude in POS erroneous response trials and incidental recall performance for negative words. Panel **(C)** illustrates the analogous association between the Pe amplitude in NEU erroneous response trials and incidental recall performance for negative words. NEG, negative; NEU, neutral; POS, positive.

To further check whether the association between incidental recall and post-error brain activity is specific to the negative condition, we conducted additional correlation analyses of memory performance for negative nouns with the Pe amplitude in general (averaged across three Error conditions), as well as with the Pe amplitude in the POS/NEU Error trials. The analyses revealed significant correlation between incidental recall for negative words and the Pe amplitude both in all erroneous response trials (*r* = 0.33, *p* = 0.012) and in the NEU Error trials (*r* = 0.30, *p* = 0.023). No correlation was found between memory performance for negative nouns and the Pe amplitude in the POS Error trials (*r* = 0.26, *p* = ns). **Figure 4** presents scatterplots illustrating how the Pe amplitude in erroneous response trials, as well as in the positive and neutral error conditions was associated with incidental recall for negative words. Pearson’s correlations were also computed to test for possible associations between ERN amplitude and memory processing. These analyses did not reveal any significant correlation between the ERN and incidental recall, either within or across emotion categories (all *p*_s_ = ns). **Table 4** presents the correlation matrix for both response-related components and memory performance for emotional and neutral stimuli^2^.

**Table 4:** Pearson correlation matrix for ERP components’ amplitude and memory performance.

		ERN time window					Pe time window				
			
		Hit	Error	NEG Error	POS Error	NEU Error	Hit	Error	NEG Error	POS Error	NEU Error
Memory	NEG	0.13	0.10	0.07	0.04	0.17	-0.09	**0.33^∗^**	**0.35^∗∗^**	0.26	**0.30^∗^**
performance	POS	0.03	0.04	0.07	-0.04	0.06	0.03	0.03	0.06	-0.04	0.05
(log-transformed)	NEU	0.01	-0.13	-0.11	-0.06	-0.17	-0.06	0.10	0.06	0.16	0.04


*Error, unsuccessfully inhibited responses; Hit, correct responses to go stimuli; NEG, negative; NEU, neutral; POS, positive. Significant effects are indicated in bold: ^∗∗^*p* < 0.01, ^∗^*p* < 0.05.*

### Exploratory Analyses

#### Correlation Analyses Between Recall Performance and Source Activation of the Pe

Numerous studies using dipole modeling or low resolution electromagnetic tomography (LORETA; Pascual-Marqui et al., 1994) have revealed that the Pe may be generated by multiple neuronal sources, encompassing the anterior cingulate, the midcingulate and posterior cingulate cortex, and additional sources in the insula, orbitofrontal and superior parietal cortex (van Veen and Carter, 2002; Herrmann et al., 2004; van Boxtel et al., 2005; Mathewson et al., 2005; O’Connell et al., 2007; Vocat et al., 2008; Dhar et al., 2011; Paul et al., 2017). This raises the question of which regions of the brain that contribute to the Pe generation are potentially involved in memory enhancement for negative words. To answer this question, we evaluated voxel-based Pearson’s correlations between the source activation of the Pe component in the NEG Error trials, measured using standardized LORETA (sLORETA; Pascual-Marqui, 2002), and incidental recall for negative words. To obtain a more detailed picture of possible associations, Pearson’s correlations were also calculated between memory performance for positive/neutral words and sLORETA source activation for the Pe in the POS/NEU Error trials respectively. Further correlation analyses were performed between incidental recall for negative words and sLORETA source activation for the Pe in erroneous response trials, as well as in the POS/NEU Error trials.

In sLORETA, computations are made in a realistic head model (Fuchs et al., 2002), using the MNI 152 template (Brain Imaging Centre, Montreal Neurologic Institute; Mazziotta et al., 2001), with the three-dimensional solution space, restricted to cortical gray matter and hippocampi. The intracerebral volume is partitioned in 6,239 voxels at 5 mm spatial resolution. Neuronal activity is computed as current density (μA/mm^2^) without assuming a predefined number of active sources. The localization accuracy of sLORETA has received considerable validation from studies combining LORETA with other methods, such as structural magnetic resonance imaging (MRI; Worrell et al., 2000), functional MRI (Vitacco et al., 2002; Mulert et al., 2004; Olbrich et al., 2009) and positron emission tomography (Dierks et al., 2000; Pizzagalli et al., 2004; Zumsteg et al., 2005). It is worth noting that even deep structures such as the anterior cingulate cortex (ACC; Pizzagalli et al., 2001) can be correctly localized with this method.

In order to identify neural correlates of memory performance, the log-transformed power of the estimated electric current density over the Pe component’s time window (180–300 ms post-response-onset) was correlated with the log-transformed percent of correctly recalled words, within and across emotion categories. The analyses corresponded to the statistical non-parametric mapping (Holmes et al., 1996) and relied on a bootstrap method with 5,000 randomized samples. This procedure gave the exact significance thresholds regardless of non-normality and corrected for multiple comparisons. The level of significance for all of the analyses was set to *p* < 0.05 for *r*-values above 0.46.

**FIGURE 5:**
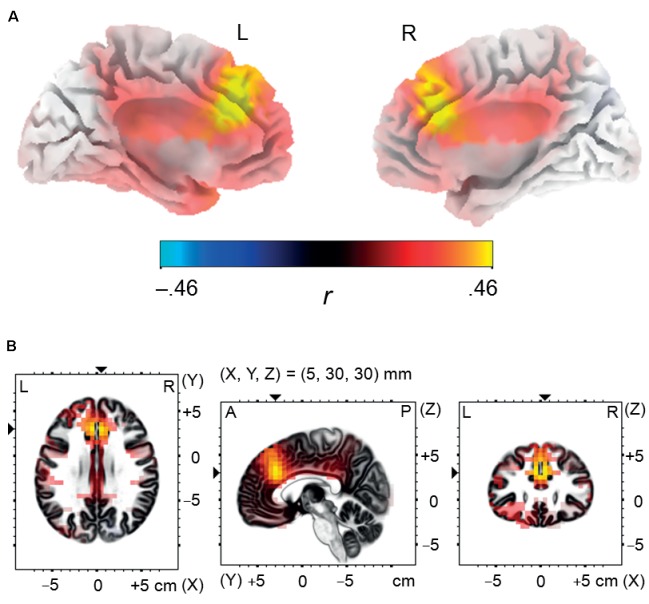
Source localization analysis. Panel **(A)** illustrates positive correlation between incidental recall performance for negative words and post-error brain activity in the dorsal anterior cingulate cortex and in the medial frontal gyrus, estimated using standardized low resolution electromagnetic tomography in the negative error trials during the time interval corresponding to the Pe component (180–300 ms post-response-onset). Panel **(B)** presents three orthogonal brain views in MNI space, sliced through the region of maximum activity. A, anterior; L, left; P, posterior; R, right.

The analysis revealed that enhanced memory performance for negative words was associated in NEG Error trials with significantly stronger activation in the bilateral network of medial frontal brain areas, encompassing the dorsal ACC and the medial frontal gyrus; see **Figure 5**. The coordinates of local maxima are provided in **Table 5**. No cortical regions displayed a significant correlation either with the percent of correctly recalled positive words (in POS Error trials) or with the percent of correctly recalled neutral words (in NEU Error trials). Moreover, no cortical regions showed a significant correlation with the percent of correctly recalled negative words either in erroneous response trials or in the POS/NEU Error trials.

**Table 5:** Brain regions showing significant Pearson correlations between memory performance for negative words and the Pe source imaging in the negative erroneous-response condition.

Brain area	Number of significant voxels	BA	Coordinates	*r*
Anterior cingulate cortex	25	32	5x, 30y, 30z	0.57
			-5x, 30y, 30z	0.55
		24	5x, 25y, 25z	0.51
			-5x, 25y, 30z	0.51
Medial frontal gyrus	18	9	5x, 30y, 35z	0.55
			-5x, 30y, 35z	0.55
		6	5x, 30y, 40z	0.52
			-5x, 30y, 40z	0.52
		8	5x, 30y, 45z	0.48
			-5x, 35y, 45z	0.49


*Maximum correlation values (*r*), coordinates of local maxima in MNI space, their respective Brodmann areas and number of significant voxels are listed for each region. BA, Brodmann area; X, Y, Z, coordinates in MNI space; X corresponds to the left–right; Y to the posterior–anterior; Z to the inferior–superior dimension.*

#### Amplitude of the P3 and the Late Positive Potential (LPP) Time-Locked to the Stop-Signal Presentation in Successfully Inhibited Trials

The ERPs time-locked to the button press in erroneous response trials and to the stop signal in unsuccessfully inhibited trials partly overlap in time due to the relatively short interval between these two kinds of events. This raises the question to what extent the erroneous-response Pe might be considered as an index of brain activation independent of that related to the stop-signal-locked P3 and LPP. This question becomes even more important as the results of previous studies suggest that emotional visual stimuli may evoke a larger P3 (e.g., Delplanque et al., 2005) and an increased LPP (e.g., Schupp et al., 2004) compared to neutral stimuli (for a review, see Olofsson et al., 2008). From this, it can be hypothesized that these two stop-signal-related positive components could be more pronounced in our study after the presentation of the emotional stop signals and could then contaminate the Pe amplitude. If the P3/LPP indeed had larger amplitudes in response to negative and positive words, the greater Pe in the emotional Error trials would not be necessarily due to the increased error monitoring, but instead to the enhanced processing of the stop signal. To rule out this possibility, we examined the amplitude of the P3 and the LPP time-locked to the stop-signal presentation in successfully inhibited trials, which are not contaminated by response-related activity^3^.

Stop-signal-related data were quantified similarly as previously described for response-related data. Stimulus-locked segments (-100 ms to 700 ms around the stop-signal onset) were aligned to the pre-stimulus baseline from -100 ms to 0 ms and averaged separately for each Stop-Signal Condition: NEG Successful Stop, POS Successful Stop and NEU Successful Stop. The mean number of correct, artifact-free epochs included in the ERP analysis across all participants for each of the stop-signal conditions was as follows: NEG Successful Stop *M* = 27.2 (*SD* = 2.7); POS Successful Stop *M* = 27.0 (*SD* = 2.7); NEU Successful Stop *M* = 26.9 (*SD* = 3.1). The minimum number of epochs was 21 for NEG Successful Stop, 21 for POS Successful Stop, 20 for NEU Successful Stop. Thus, stop-signal-related components were based on no fewer than 20 artifact-free error trials, a number that is sufficient to achieve stable estimates of the P3 (Cohen and Polich, 1997). Consistent with previous research (Zheng et al., 2011; Fritsch and Kuchinke, 2013), time windows were selected around the P3 (270–440 ms) and the LPP (440–540 ms). Mean voltage amplitudes in the component-specific windows were used for statistical analysis. In line with previously described analyses, we focused on electrode Cz. To analyze the amplitudes of the P3 and LPP, one-way repeated-measures ANOVA was conducted separately for each ERP component with the Stop-Signal Condition (NEG, POS and NEU Successful Stop) as factor. The distributions of the variables were not statistically different from the normal distribution. In addition, correlation analyses were performed to examine potential associations between memory processing and both stop-signal-related components. The critical *p*-value was set at 0.05 for all the analyses.

**FIGURE 6:**
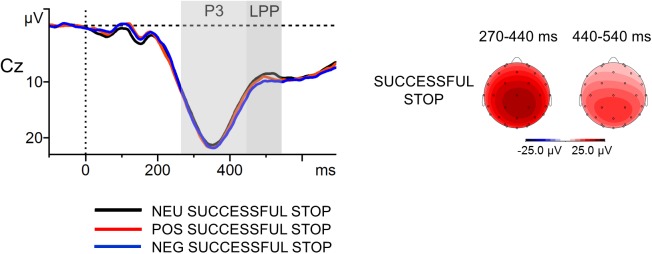
Stop-signal-locked grand-average waveforms at Cz electrode (left part) with scalp potential maps for the P3 and LPP components (right part) in successfully inhibited trials. The component-specific windows examined in this study are highlighted. NEG, negative; NEU, neutral; POS, positive.

The grand-average ERPs to the stop signal at Cz with scalp distribution maps in successfully inhibited trials are presented in **Figure 6**. The first ANOVA revealed that the main effect of Stop-Signal Condition was not significant in the P3 time window [*F*(2,114) = 1.25, *p* = ns], contrary to the results obtained for Pe and Error Condition. The P3 amplitudes were statistically comparable in the NEG (*M* = 18.3 μV, *SD* = 7.2], POS (*M* = 18.1 μV, *SD* = 6.7) and NEU (*M* = 17.6 μV, *SD* = 8.1) Successful Stop trials. The second ANOVA showed only a weak trend toward an effect of Stop-Signal Condition for the LPP [*F*(2,114) = 2.69, *p* = 0.07, $\eta_{p}^{2}$ = 0.05]. The mean voltage amplitudes observed in the LPP-specific window were as follows: NEG Successful Stop *M* = 10.6 (*SD* = 5.4); POS Successful Stop *M* = 10.0 (*SD* = 4.8); NEU Successful Stop *M* = 9.4 (*SD* = 6.2). Moreover, correlation analyses did not reveal any significant association between stop-signal-related components and memory processing, either within or across emotion categories (all *p*_s_ = ns).

Thus, the correct-stop P3 and LPP observed on Cz electrode were not substantially larger in the emotional than in the neutral stop-signal trials. They were also not associated with incidental recall for words. This pattern of results suggests that the within-condition difference in the Pe amplitude was indeed generated by error-related processes. Therefore, it seems safe to conclude that in the SST the erroneous-response Pe reflect functionally distinct aspect of cognitive control from that associated with stop-signal-locked positivities.

## Discussion

The present study had three main objectives. First, we intended to test whether short-duration affective states induced by unpleasant and pleasant nouns can lead to increased error-monitoring activity relative to a condition involving neutral nouns. Second, we aimed to check whether such an enhancement is limited to words of specific valence or is a general response to arousing material. Third, we wanted to assess whether post-error brain activity can support incidental memory for negative and/or positive words. Our initial hypothesis that error monitoring would be enhanced in the emotional conditions was confirmed. In particular, we found significantly larger error-related brain activity in the Pe time window in both negative and positive trials. Regarding behavior, enhanced processing of negative and positive words was reflected in better incidental memory. Moreover, we observed that memory performance for negative words was positively correlated with the Pe amplitude, especially in the negative condition. Following up on this correlation, we performed source localization analysis in order to estimate the neural correlates of this effect. The results of sLORETA analysis revealed that the memory recall for negative words was associated with widespread bilateral activations in the anterior cingulate gyrus and in the medial frontal gyrus.

### Emotional Enhancement of Error Monitoring

The analyses revealed that both error-related ERP components, namely ERN and the Pe, were more pronounced in erroneous than in correct response trials; this corresponds with previous research (Falkenstein et al., 1991; Nieuwenhuis et al., 2001). We observed comparable ERN amplitudes in the neutral and in emotional arousing trials, regardless of their affective valence. This pattern of results is in line with our findings from two previous SST studies with threatening visual and aversive auditory stop signals (Senderecka, 2016, 2018). The lack of emotional modulation of the ERN points to the possibility that the post-response conflict (Botvinick et al., 2001; Yeung et al., 2004) or mismatch between the actual response and the desired state (Falkenstein et al., 1991; Coles et al., 2001) had a similar degree in all conditions of the task. This may also suggest that the increase in attentional control (van Noordt et al., 2015, 2016, 2017) or the decrease in dopaminergic activity (Holroyd and Coles, 2002) evoked by unexpected negative outcomes of an action were comparable across the three stop-signal categories. Finally, this result may also indicate that at the early stage of performance monitoring, the subjective significance of an error (Gehring et al., 1993; Hajcak et al., 2005) or the accompanying emotional distress (Hajcak and Foti, 2008; Inzlicht and Al-Khindi, 2012) did not differ between the negative, positive and neutral conditions.

Our results stand in contrast to previous works reporting ERN amplitude modulation in response to affective state induction (Larson et al., 2006; Wiswede et al., 2009a,b; van Wouwe et al., 2010; Ogawa et al., 2011; Riesel et al., 2012; Pfabigan et al., 2013). However, they align with less numerous yet informative studies that did not find such an influence (Moser et al., 2005; Paul et al., 2017). It should be underlined that the aforementioned studies are difficult to compare due to the substantial variability in methodology, including the nature of the task (flanker task, Stroop task, continuous performance task, go/no-go task or SST), the type of affect-induction procedure (based on bottom–up or top–down emotional manipulation) and finally the nature of the errors (errors in choice-reaction tasks or inhibition errors). Thus, these contradictory or at least equivocal findings may be attributed to specific procedure demands, and certainly call for further investigations to elucidate the influence of short-duration affective states on early stages of error monitoring.

As for the second stage of error processing represented by the Pe, a clear pattern of emotional modulation was apparent. The Pe amplitude was larger in the negative and positive trials than in the neutral ones. In the literature, the Pe has generally been associated with conscious appraisal of erroneous responses (Nieuwenhuis et al., 2001; Endrass et al., 2007; Larson and Perlstein, 2009; Hughes and Yeung, 2011) and the motivational significance of an error (Leuthold and Sommer, 1999; Ridderinkhof et al., 2009; Endrass et al., 2012). Thus, a possible interpretation of our findings may be that participants were more aware of the errors committed after the presentation of the emotional stop signals. Therefore, these errors might have been more motivationally salient and attentionally engaging for them. Alternatively, the larger Pe in the emotional trials might also have reflected an enhanced affective appraisal of errors (Falkenstein et al., 2000). One additional possibility of considerable interest is that the larger Pe reflected an enhanced accumulation of evidence that an error had occurred (Steinhauser and Yeung, 2010).

It is worth noting that these different accounts of the ERN and Pe are not mutually exclusive. Rather, they emphasize different aspects of the cognitive-emotional system responsible for goal-directed behavior. Thus, we do not purport to adjudicate between these models with our present data. Instead, we accept that there are several plausible interpretations to explain enhanced Pe amplitudes in emotional conditions of our task.

The present results, in conjunction with our previous studies (Senderecka, 2016, 2018), demonstrate that the emotional amplification of the Pe amplitude occurs across a variety of affective stimulus types. Thus, the enhancement of error monitoring evoked by task-relevant, affective material is not restricted to stimuli with evolutionary significance (e.g., threatening pictures or aversive sounds), but instead extends to material with symbolic, ontogenetically learned emotional significance. Moreover, our analyses revealed that highly arousing words from two emotional valence categories modulated the Pe amplitude in a similar way. Hence, this pattern of results indicates that the affective enhancement of error monitoring that occurs across both negative and positive conditions is preferentially driven by the arousal content of an emotional stimulus.

The present data also suggest that various components of the error processing system are differentially sensitive to diverse emotional manipulation. Given that ERN and Pe are thought to reflect independent aspects of post-error processing, with the former primarily linked to conflict monitoring (Botvinick et al., 2001; Yeung et al., 2004) and the latter preferentially associated with conscious error recognition and remedial action (Nieuwenhuis et al., 2001; Endrass et al., 2007; Larson and Perlstein, 2009; Hughes and Yeung, 2011), such results are not surprising. Considering results from previous studies, it seems reasonable to tentatively assume that ERN may be primarily modulated by trait-related affective dispositions (Vaidyanathan et al., 2012; Endrass and Ullsperger, 2014) and is relatively less sensitive to short-duration affective states induced by emotional stimuli. Simultaneously, a growing body of evidence indicates that the Pe amplitude is state-dependent and may be reliably modulated by affective stimuli presentation (Moser et al., 2005; Senderecka, 2016, 2018; Paul et al., 2017). Further research is surely needed to attain a thorough understanding of the associations between emotional states and the variability of these two error-related components.

### Links Between Post-error Brain Activity and Incidental Memory

The behavioral outcomes of the SST revealed that emotional words did not influence the stop-signal reaction time and inhibitory rate, as compared to neutral words. This may suggest that the arousing power of the linguistic material was not sufficient to interfere with inhibitory performance, similarly to what has been observed in other tasks that target various cognitive functions (e.g., Williams et al., 1996; Siegle et al., 2002). However, although participants did not have to explicitly process the meaning of the words during SST, the emotional arousal effect of linguistic stimuli was seen in incidental recall. Both negative and positive nouns produced a benefit in memory performance as compared to neutral nouns, this is in line with the EEM effect observed in previous studies (for a review, see Murty et al., 2010). Participants were not forewarned of the subsequent free recall test, thus any effect observed on the recall should be attributable to incidental learning during encoding.

The general memory improvement for affective material could be due to multiple factors that play a significant role during encoding, such as greater attentional engagement (Hamann, 2001; Calvo and Lang, 2004), enhanced perceptual sensitivity (Zeelenberg et al., 2006), or increased physiological arousal (LeDoux, 2000) in response to emotional stimuli. They can be associated with greater activation of the amygdala, hippocampus, frontal and temporal cortices, as well as the ventral visual stream during encoding (Murty et al., 2010). Altogether, these different mechanisms provide multiple, additive or interactive sources of modulation for the processing of emotional stimuli that ultimately determine their privileged access to awareness and memory systems. Thus, all these factors possibly contributed to the memory enhancement for negative and positive words in the present study.

Additionally, our analyses revealed an interesting correlation of memory performance for negative words with the Pe amplitude across all erroneous trials, as well as in the negative and neutral conditions in particular. Since our study was correlational in nature, no strong conclusions can be drawn about the exact mechanism of these effects. However, at least two interpretations can be offered to explain our findings. First, our results provide evidence that enhanced error monitoring is associated with facilitated recall of emotionally negative words that have been encoded during the experimental session. However, this correlation does not necessarily reveal cause and effect relationship. Enhanced error monitoring and facilitated recall of negative words may co-occur because they both reflect responsivity to negative information^4^. Errors are maladaptive reactions that may put an individual in danger, whereas negative words are symbolic representations of concepts, places, or objects that are likely to threaten his or her safety. Since both errors and unpleasant linguistic stimuli are negative events, individuals who are especially sensitive to their own errors might also be particularly inclined to allocate more attention to negative words, improving their encoding and subsequent recall from memory (Hamann, 2001; Calvo and Lang, 2004; Talmi and McGarry, 2012). Consequently, in our study, these participants who showed larger Pe amplitudes could recall more negative nouns. No such association was observed between the Pe amplitude and incidental recall for positive and neutral nouns, because errors and words from two other categories were not emotionally congruent events. The non-causal relation between enhanced error monitoring and facilitated recall of negative words is additionally supported by the fact that memory performance for negative words was significantly correlated not only with the Pe amplitude in the negative condition but also with the Pe amplitude across all erroneous trials and in the neutral condition. Thus, it can be assumed that memory improvement for negative material and an increased amplitude of the Pe were associated with each other because they are both related to individual differences in emotionality. That is, individuals who are characterized by high sensitivity to negative events exhibit enhanced recall of unpleasant words and increased error monitoring, as indexed by the Pe amplitude.

Alternatively and more speculatively, our findings might also be interpreted as indicating a causal link between post-error brain activity and enhanced recall of negative words. This second interpretation relies on the results of source localization analysis. It revealed that enhanced recall of negative words correlated positively with the brain activity in the dorsal ACC and in the dorsomedial prefrontal cortex (dmPFC) that was registered in the Pe time window during negative trials. No such correlation was observed between memory performance for negative words and medial prefrontal brain activity during positive, neutral or globally erroneous trials. This specific pattern of results suggests that error-related brain activity in the negative condition may selectively support memory encoding for negative material. The ACC and the dmPFC are known to be engaged in both cognitive and affective processing (Phan et al., 2004). The dorsal ACC contributes to error monitoring (for a review, see Ridderinkhof et al., 2004), but can also act as salience detector when faced with emotional stimuli (Davis et al., 2005). Moreover, both the dorsal ACC and the dmPFC are strongly activated during fear conditioning (Etkin et al., 2011). These activations probably reflect threat appraisal, accompanied by learning processes. Although the exact brain activity elicited during error monitoring and threat appraisal within learning processes may differ significantly, they nonetheless involve at least partially overlapping neural networks. Therefore, it seems reasonable to tentatively assume that the neural processes involved in error detection in the negative condition may have a facilitative effect on the neural processes underlying unpleasant stimuli encoding due to an overlap of the neural networks behind these two functions. Within this interpretation, the EEM effect observed for positive words could be based on a different neural mechanism, probably related to enhanced perceptual sensitivity (Zeelenberg et al., 2006) or increased physiological arousal (LeDoux, 2000), but operating independently of the error monitoring process.

## Conclusion and Future Directions

Using error-related ERP components and behavioral measures, this study examined the links between short-duration affective states induced by emotional nouns, error monitoring, and incidental memory. In particular, we investigated, first, whether emotional words can lead to increased error monitoring, as reflected by the ERN and Pe amplitudes, relative to a neutral task condition, and second, whether this enhancement can be differentially modulated by affective valence. Our third goal was to assess whether post-error brain activity is associated with incidental memory for negative and/or positive words.

According to our hypothesis, we found significantly larger error-related brain activity in the Pe time window in both negative and positive conditions. In contrast, the ERN amplitudes were comparable in all types of trials, regardless of their affective valence. These findings suggest that the emotional enhancement of error monitoring, as reflected by the Pe amplitude, may be induced by stimuli with symbolic, ontogenetically learned emotional significance. They also indicate that the emotion-related enhancement of the Pe is not limited to words of specific valence, thus, it is preferentially driven by the arousal content of an affective stimuli. Moreover, they provide additional evidence that the ERN and Pe reflect independent aspects of post-error processing and are differentially sensitive to emotional manipulation.

Importantly, to our knowledge, this is the first study that has examined memory performance in an error-monitoring context. In correspondence with the EEM effect described in previous studies, we observed that both negative and positive nouns produced a benefit in incidental recall as compared to neutral nouns. Interestingly, the memory performance for negative words turned out to be positively correlated with the Pe amplitude, particularly in the negative condition. The sLORETA analysis revealed that the subsequent memory recall for negative words was associated with widespread bilateral activations in the dorsal anterior cingulate cortex and in the medial frontal gyrus, registered in the Pe time window during negative trials. These results suggest that enhanced error monitoring and facilitated recall of negative words may both reflect responsivity to negative events. More speculatively, they can also indicate that post-error activity of the medial prefrontal cortex may selectively support encoding for negative stimuli and contribute to their privileged access to memory.

Some limitations of the present work and future directions for research should be mentioned here. First, although LORETA is a widely used and empirically well-supported source localization method (Pascual-Marqui et al., 1994; Pascual-Marqui et al., 2002), the inverse solution results should be always interpreted with caution because of the imprecise nature of the mathematical reconstruction on which they are based. In addition, the present results were obtained using small number (32) of scalp electrodes, thus, they must be considered with reservation. With low spatial resolution, there is a decreased chance that LORETA will be able to effectively identify the closely spaced sources (Greenblatt et al., 2005). Further research is surely needed to validate our findings by using an enhanced spatial resolution.

Second, in studies with long retention intervals the EEM probably relies on a different mechanism than in tasks involving a short delay between an initial encoding and subsequent recall (Talmi et al., 2007). In the former case, the EEM is primarily due to a better consolidation of emotional memory traces. Taking into account this divergence, further research is necessary to determine whether post-error brain activity is associated with memory performance when tested after a long delay.

Third, in the present study a large set of emotional and neutral nouns selected from a standardized database was used to induce short-duration affective states. Therefore, the choice of the linguistic stimuli precluded the examination of whether the observed association between error monitoring and memory performance can be observed for other kinds of aversive stimuli. Thus, it would surely be worthwhile to replicate the present results using emotional pictures, which from an evolutionary perspective are more biologically salient and motivationally relevant than words.

## Author Contributions

MS developed the rationale for the study, designed the experiment, analyzed the data, and wrote the manuscript. MO prepared the experimental task. MM collected the data. BK contributed analysis tools for behavioral data. All authors reviewed the manuscript.

## Conflict of Interest Statement

The authors declare that the research was conducted in the absence of any commercial or financial relationships that could be construed as a potential conflict of interest.

## Footnotes

1 The correlation remained significant even when one participant with unusually high percentage (40.7%) of correctly recalled negative words was excluded from the analysis (*r* = 0.33, *p* = 0.011).

2 The analogous Spearman rank correlation coefficients for both response-related components and percentages of correctly recalled negative, positive and neutral words (without applying the log-transformation) are presented in Supplementary Table S2. The results are mainly in line with those obtained using Pearson’s correlations on the log-transformed data.

3 We thank the Reviewers for drawing these analyses to our attention.

4 We thank the Reviewers for directing our attention to this interpretation.
