# Supplementary material for: Post-error Brain Activity Correlates With Incidental Memory for Negative Words
Source: Front Hum Neurosci. 2018 May 8;12:178. doi: 10.3389/fnhum.2018.00178 (PMC5951961; doi:10.3389/fnhum.2018.00178)
Supplement: Supplementary file 1 [file Table_1.pdf]

Supplementary Material

Post-Error Brain Activity Correlates with Incidental Memory for Negative Words

Magdalena Senderecka*, Michał Ociepka, Magdalena Matyjek, Bartłomiej Kroczek

*** Correspondence:**
Magdalena Senderecka
E-mail: magdalena.senderecka@uj.edu.pl

TABLE S1 | NAWL words used in the study

**Negative Words**	alkoholik, awantura, ból, gniew, kara, katastrofa, kłamstwo, kłótnia, koszmar, lęk, morderstwo, napad, niepokój, oszust, panika, przemoc, samobójstwo, strach, śmierć, tortura, trup, wojna, wypadek, zabójca, zemsta, złodziej, zwłoki
**Neutral Words**	archiwum, bateria, cecha, dąb, dokument, dźwignia, fasada, instytucja, lada, leksykon, lista, makulatura, nos, obiekt, obszar, odbitka, płaszcz, podłoże, posiedzenie, produkt, przedział, segregator, sylaba, sztućce, teczka, wiadro, worek
**Positive Words**	awans, bliskość, cud, geniusz, hobby, mądrość, miłość, odwaga, prezent, przyjaciel, przyjemność, radość, raj, seks, spełnienie, sukces, szansa, szczęście, urlop, urodziny, uśmiech, wakacje, weekend, wierność, wiosna, wolność, zwycięstwo
