# Supplementary material for: Post-error Brain Activity Correlates With Incidental Memory for Negative Words
Source: Front Hum Neurosci. 2018 May 8;12:178. doi: 10.3389/fnhum.2018.00178 (PMC5951961; doi:10.3389/fnhum.2018.00178)
Supplement: Supplementary file 2 [file Table_2.pdf]

Supplementary Material

Post-Error Brain Activity Correlates with Incidental Memory for Negative Words

Magdalena Senderecka*, Michał Ociepka, Magdalena Matyjek, Bartłomiej Kroczek

*** Correspondence:**
Magdalena Senderecka
E-mail: magdalena.senderecka@uj.edu.pl

TABLE S2 | Spearman correlation matrix for ERP components' amplitude and memory performance

		ERN time window					Pe time window				
		Hit	Error	NEG Error	POS Error	NEU Error	Hit	Error	NEG Error	POS Error	NEU Error
Memory performance (percentages)	NEG	0.03	0.14	0.05	0.06	0.19	-0.14	**0.28***	**0.28***	0.22	**0.31***
	POS	0.03	0.04	0.09	-0.03	0.06	-0.06	-0.02	0.05	-0.10	0.04
	NEU	0.04	-0.09	-0.10	-0.04	-0.15	-0.03	0.11	0.07	0.14	0.08

Note. Error, unsuccessfully inhibited responses; Hit, correct responses to go stimuli; NEG, negative; NEU, neutral; POS, positive. Significant effects are indicated in bold: * $p < 0.05$.
